# Supplementary material for: Escalating progression of mental health disorders during the COVID-19 pandemic: Evidence from a nationwide survey
Source: PLoS One. 2021 Mar 25;16(3):e0248916. doi: 10.1371/journal.pone.0248916 (PMC7993793; doi:10.1371/journal.pone.0248916)
Supplement: Attachment — Submitted filename: Response to reviewers 02.03.2021.docx [file pone.0248916.s001.docx]

Journal Requirements:

When submitting your revision, we need you to address these additional requirements.

1. Please ensure that your manuscript meets PLOS ONE's style requirements, including those for file naming. The PLOS ONE style templates can be found at

<https://journals.plos.org/plosone/s/file?id=wjVg/PLOSOne_formatting_sample_main_body.pdf> and

https://journals.plos.org/plosone/s/file?id=ba62/PLOSOne_formatting_sample_title_authors_affiliations.pdf

2.  We note that you have indicated that data from this study are available upon request. PLOS only allows data to be available upon request if there are legal or ethical restrictions on sharing data publicly. For more information on unacceptable data access restrictions, please see <http://journals.plos.org/plosone/s/data-availability#loc-unacceptable-data-access-restrictions>.

 In your revised cover letter, please address the following prompts:

 a) If there are ethical or legal restrictions on sharing a de-identified data set, please explain them in detail (e.g., data contain potentially sensitive information, data are owned by a third-party organization, etc.) and who has imposed them (e.g., an ethics committee). Please also provide contact information for a data access committee, ethics committee, or other institutional body to which data requests may be sent.

Reply: We do not have ethical or legal restrictions on sharing a de-identified data set, we noted in the cover letter. We have also uploaded our data in data repository Havard dataverse (<https://doi.org/10.7910/DVN/COITOK>)

All data files are available from the Havard dataverse (accession number(s) <https://doi.org/10.7910/DVN/COITOK>).

b) If there are no restrictions, please upload the minimal anonymized data set necessary to replicate your study findings as either Supporting Information files or to a stable, public repository and provide us with the relevant URLs, DOIs, or accession numbers. For a list of acceptable repositories, please see <http://journals.plos.org/plosone/s/data-availability#loc-recommended-repositories>.

Reply: The data is uploaded in the following data repository Havard dataverse

Link database: <https://doi.org/10.7910/DVN/COITOK>

 We will update your Data Availability statement on your behalf to reflect the information you provide.

3. Please include captions for your Supporting Information files at the end of your manuscript, and update any in-text citations to match accordingly. Please see our Supporting Information guidelines for more information: http://journals.plos.org/plosone/s/supporting-information.

 Reply: Added

[Note: HTML markup is below. Please do not edit.]

Reviewers' comments:

Reviewer's Responses to Questions

**Comments to the Author**

1. Is the manuscript technically sound, and do the data support the conclusions?

The manuscript must describe a technically sound piece of scientific research with data that supports the conclusions. Experiments must have been conducted rigorously, with appropriate controls, replication, and sample sizes. The conclusions must be drawn appropriately based on the data presented.

Reviewer #1: Yes

2. Has the statistical analysis been performed appropriately and rigorously?

Reviewer #1: Yes

3. Have the authors made all data underlying the findings in their manuscript fully available?

The [PLOS Data policy](http://www.plosone.org/static/policies.action#sharing) requires authors to make all data underlying the findings described in their manuscript fully available without restriction, with rare exception (please refer to the Data Availability Statement in the manuscript PDF file). The data should be provided as part of the manuscript or its supporting information, or deposited to a public repository. For example, in addition to summary statistics, the data points behind means, medians and variance measures should be available. If there are restrictions on publicly sharing data—e.g. participant privacy or use of data from a third party—those must be specified.

 Data has been deposited in data repository (<https://doi.org/10.7910/DVN/COITOK>)

Reviewer #1: Yes

4. Is the manuscript presented in an intelligible fashion and written in standard English?

PLOS ONE does not copyedit accepted manuscripts, so the language in submitted articles must be clear, correct, and unambiguous. Any typographical or grammatical errors should be corrected at revision, so please note any specific errors here.

Reviewer #1: Yes

5. Review Comments to the Author

Please use the space provided to explain your answers to the questions above. You may also include additional comments for the author, including concerns about dual publication, research ethics, or publication ethics. (Please upload your review as an attachment if it exceeds 20,000 characters)

Reviewer #1: General comment:
The manuscript entitled “Escalating progression of mental health disorders during the COVID-19 pandemic: evidence from a nationwide survey” highlighted the mental health concerns of the Malaysian population in the COVID-19 pandemic. The strength of the manuscript is the timely assessment during COVID-19 with large sample size. However, some parts of the manuscript should be elucidated and clarified.
Mainly, different MCOs was introduced, yet the purpose of this introduction was not clear. Furthermore, the rationale of dividing four time periods was not explained well. Is it related to the MCOs, or due to other reasons? Please see the specific comments for details.


Specific comments:

Introduction
(1) For the first paragraph, “The first MCO included the closure of schools, higher education institutions and ‘non-essential’ businesses”.
Why quotation mark was used? Is there any special meaning for the non-essential? Authors may provide examples for the non-essential businesses.

Reply: We remove ‘ ‘

Added: meaning of non-essential businesses, line 72-74

⇒The first MCO included the closure of schools, higher education institutions and non-essential businesses (namely businesses that geared toward recreation or entertainment and those that provide services beyond the basic necessities),……..

(2) Authors introduced different MCOs in the first paragraph. Is the author simply tried to illustrate the situation in Malaysia, or tried to illustrate the impact of COVID-19 and related MCOs?
If authors think MCOs is important in this study, they should discuss the results incorporate with MCOs. For example, authors mentioned RMCO had more lenient restrictions compared to CMCO. Should we expect the negative impact on people’s daily living was reduced under RMCO, and hence, may influence mental health?

Reply: Thank you for highlighting the shortcoming. In line 135-6, we noted “The temporal trend of the DASS-21-D, DASS-21-A and DASS-21-S scores over the 16-week data collection period was computed”. Hence we added in line 98 the objective of investigating the temporal trend of mental health.

⇒Therefore, the main aim of this study was to examine the level and temporal trend of mental health of the Malaysian public during the COVID-19 pandemic.

In Introduction, we provide a snapshots of the different phases of MCOs in Malaysia, however, the data collection was conducted after during CMCO and RMCO, where the government imposed more lenient containment measures.

Added, although ideally when restrictions were lifted, mental health improve, but out study found otherwise. The possible reasons were noted in line 245-252.

Added line 242-245

⇒Although psychological well-being of the public is expected to improve when restrictions were lifted, nonetheless, the negative mental impact of the people in this study did not decline despite the shift from CMCO to RMCO. The psychological impact continues to rise across the CMCO and RMCO phase.

Materials and methods
(1) Any exclusion criteria for the recruitment?

Reply: Added exclusion criteria, line 108

⇒The exclusion criteria were as follow: having chronic medical conditions, pregnancy or breastfeeding, and have never had SARS-CoV-2 infection.

(2) “The participants were asked to indicate whether they know of friends, neighbours or colleagues who had been diagnosed with COVID-19.”
How about the participants themselves? Whether participants had been diagnosed with COVID-19 might be important to their mental health.

Reply: The study did not include people who have had been diagnosed with COVID-19 as noted in the added exclusion criteria.

(3) In this study, both English version and Bahasa Malaysia version of DASS-21 were used and pooled for analysis. Could authors provide reference to support the measurement invariance across these two language versions?

Reply: To our best knowledge, to date, there is no reference to support the measurement invariance across the English and Bahasa Malaysia version of the DASS-21. Unfortunately, in our study, the English and Bahasa Malaysia questions of the DASS-21 were place side by side and hence the measurement of invariance across the English and BM versions of the DASS-21 is unable to be calculated in the current study.

We reported the limitation of the measurement invariance across these two language versions.

Added line 329
⇒It is also important to note that this study used both the English and the Bahasa Malaysia version of DASS-21, however, the measurement invariance across the English and Bahasa Malaysia version of DASS-21 was unable to be determined as both version of the DASS-21 were included in the same survey link.

Data analysis

(1) I suggest to report internal consistency for English version and Bahasa Malaysia version separately.

Reply: Thank you for suggestion, unfortunately in our survey, both Bahasa Malaysia and English questions were incorporated into our sole survey link. Each questions is bilingual, hence we are unable to report internal consistency for both version. We are unsure if the respondent were reading the English or BM version when answering the questions.

(2) Could authors explain the rationale of dividing four time periods? Without any explanation, it made me wonder whether it was related to the MCOs. However, it seems that they did not match.

Reply: The four time periods were divided by equal time intervals. We introduces the phases of MCO, CMCO and RMCO in the Introduction to provide information of all stages of movement control in Malaysia during the pandemic, however, our data collection was conducted during CMCO and RMCO. Hence, rational of dividing to four time periods was not the sequence of the MCO, CMCO and RMCO. We could only relate the findings on the two phases of MCOs.

Added “by equal intervals”, hence added the word “equal” line line 138

⇒The mean and standard deviation (SD) of the DASS-21 subscale scores were divided into four equal time periods of 4-week intervals….

Results and discussion
(1) As the study divided four time periods, the number of participants in each period should be provided.

Reply: We noted the number of participants in each phases in Fig. 2. We made minor typo mistakes on the number of responses, corrected them in text and tables, and shown in the correction tracking. The rest of the information is accurate.

We added in the limitation of the low number of responses in the last two time periods. Line 332.

⇒Lastly, the study is also limited by the small number of responses in the last two time periods, hence results should be interpreted with caution.

(2) For those who reported their gender, most of them were female. Authors may consider to discuss it in the limitation.

Added in line 327.

⇒The study also has a higher representation of female participants.

6. PLOS authors have the option to publish the peer review history of their article ([what does this mean?](https://journals.plos.org/plosone/s/editorial-and-peer-review-process#loc-peer-review-history)). If published, this will include your full peer review and any attached files.


If you choose “no”, your identity will remain anonymous but your review may still be made public.


**Do you want your identity to be public for this peer review?** For information about this choice, including consent withdrawal, please see our [Privacy Policy](https://www.plos.org/privacy-policy).

Reviewer #1: No

[NOTE: If reviewer comments were submitted as an attachment file, they will be attached to this email and accessible via the submission site. Please log into your account, locate the manuscript record, and check for the action link "View Attachments". If this link does not appear, there are no attachment files.]

While revising your submission, please upload your figure files to the Preflight Analysis and Conversion Engine (PACE) digital diagnostic tool, <https://pacev2.apexcovantage.com/>. PACE helps ensure that figures meet PLOS requirements. To use PACE, you must first register as a user. Registration is free. Then, login and navigate to the UPLOAD tab, where you will find detailed instructions on how to use the tool. If you encounter any issues or have any questions when using PACE, please email PLOS at [figures@plos.org](mailto:figures@plos.org). Please note that Supporting Information files do not need this step.
