# Supplementary material for: Escalating progression of mental health disorders during the COVID-19 pandemic: Evidence from a nationwide survey
Source: PLoS One. 2021 Mar 25;16(3):e0248916. doi: 10.1371/journal.pone.0248916 (PMC7993793; doi:10.1371/journal.pone.0248916)
Supplement: Attachment — Submitted filename: Response to Reviewers 5March 2021.docx [file pone.0248916.s002.docx]

In general, the responses are decent and I only have one concern regarding the measurement invariance.

Apparently, the authors cannot justify why the two language versions can be combined used and thus they put this into their limitation. I think that putting this as one of the limitations is appropriate. However, the authors should emphasize this issue and encourage future studies to investigate the measurement invariance.

Reply: We emphasized the importance of the issue and the importance of future studies to investigate the measurement invariance.

⇒Testing for and assuring measurement invariance across different languages, culture or group comparisons is essential [42-44]. Future studies on DASS-21 should include validation across different group comparisons and testing of invariance of all versions.

The authors should also mention why it is important to investigate measurement invariance with proper citation. They can take reference from Leung et al. (2020).Leung, H., Pakpour, A. H., Strong, C., Lin, Y. C., Tsai, M. C., Griffiths, M. D., Lin, C. Y., & Chen, I. H. (2020). Measurement invariance across young adults from Hong Kong and Taiwan among three internet-related addiction scales: Bergen Social Media Addiction Scale (BSMAS), Smartphone Application-Based Addiction Scale (SABAS), and Internet Gaming Disorder Scale-Short Form (IGDS-SF9) (Study Part A). Addictive behaviors, 101, 105969. <https://doi.org/10.1016/j.addbeh.2019.04.027>

Reply: We noted that the importance of investigating measurement invariance to ensure psychometric robustness.

⇒ In the absence of measurement invariance procedures across the English and Bahasa Malaysia version of DASS-21 in this study, the psychometric robustness associated with different interpretations of items in the two languages was unknown.

Reply: We rephrased the limitation section as follow.

To our best knowledge, the measurement of invariance across the English and Bahasa Malaysia version of DASS-21 has never been examined before. Unfortunately, the measurement invariance across the English and Bahasa Malaysia version of DASS-21 was unable to be determined in our bilingual survey link. In the absence of measurement invariance procedures across the English and Bahasa Malaysia version of DASS-21 in this study, the psychometric robustness associated with different interpretations of items in the two languages was unknown. Testing for and assuring measurement invariance across different languages, culture or group comparisons is essential [42-44]. Future studies on DASS-21 should include validation across different group comparisons and testing of invariance of all versions.
